# Genome Writing: Current Progress and Related Applications

**DOI:** 10.1016/j.gpb.2018.02.001

**Published:** 2018-02-21

**Authors:** Yueqiang Wang, Yue Shen, Ying Gu, Shida Zhu, Ye Yin

**Affiliations:** 1 BGI-Shenzhen, Shenzhen 518083, China; 2 China National GeneBank, BGI-Shenzhen, Shenzhen 518083, China; 3 BGI-Qingdao, Qingdao 266555, China; 4 Shenzhen Engineering Laboratory for Innovative Molecular Diagnostics, BGI-Shenzhen, Shenzhen 518083, China; 5 Guangdong Provincial Key Laboratory of Genome Read and Write, Shenzhen 518120, China; 6 BGI Genomics, BGI-Shenzhen, Shenzhen 518083, China; 7 School of Life Science and Biotechnology, Dalian University of Technology, Dalian 116023, China; 8 Laboratory of Genomics and Molecular Biomedicine, Department of Biology, University of Copenhagen, Copenhagen 2100, Denmark

**Keywords:** Synthetic biology, Genome writing, Genome editing, Bioethics, Biosafety

## Abstract

The ultimate goal of **synthetic biology** is to build customized cells or organisms to meet specific industrial or medical needs. The most important part of the customized cell is a synthetic genome. Advanced genomic writing technologies are required to build such an artificial genome. Recently, the partially-completed synthetic yeast genome project represents a milestone in this field. In this mini review, we briefly introduce the techniques for *de novo* genome synthesis and **genome editing**. Furthermore, we summarize recent research progresses and highlight several applications in the synthetic genome field. Finally, we discuss current challenges and future prospects.

## Introduction

**Figure 1:**
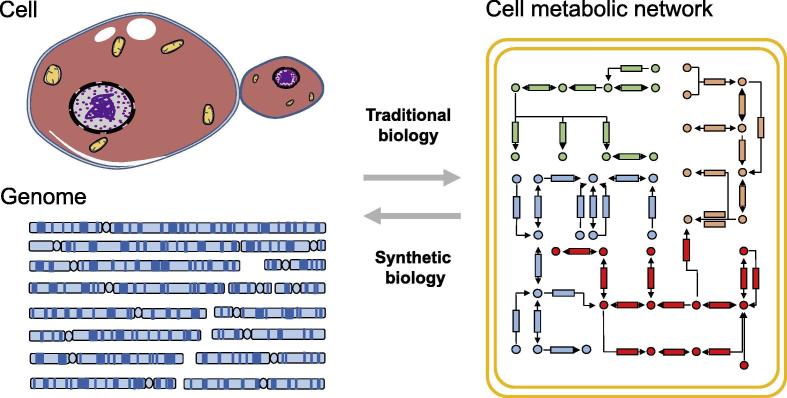
**Relation between traditional biology and synthetic biology** Traditional biological studies aim to understand how genes in a specific genome control different cellular processes such as metabolisms. In contrast, synthetic biology involves in the design and synthesis of an artificial genome with the aim to build a cell from scratch with designed functions. The yellow rectangles with rounded corners represent the cell membrane. This cell contains a variety of metabolic pathways like those depicted in green, blue, yellow, and red, respectively. In the pathways shown, the circle represents the metabolite, the rectangle represents a specific enzyme, and the arrow represents the direction of the enzymatic reaction.

Biologists have long been focusing on understanding the natural biological processes and mechanisms. Lots of fundamental questions await to be answered, *e.g.*, how the genome regulates the entire cellular metabolic network and how unicellular organisms evolve into multicellular organisms. In this sense, one of the core questions for the conventional biological studies is how the phenotypic features of a specific organism are controlled by its genome (Figure 1). Synthetic biology, on the other hand, takes an entirely new direction to address this question by adopting a reverse approach: modify or *de novo* synthesize an organism’s genome in order to enable designed biological features. As Richard Feynman once said, “*What I cannot create, I do not understand*”. The ultimate goal of synthetic biology is to build a living cell or an organism with designed functions (Figure 1) [1]. The success of synthetic biology, in a large part, relies on a customized artificial genome. Genome writing is to build such a customized genome according to people's designs. Currently, several *de novo* synthetic genome projects are underway [2]. In parallel, in addition to building a genome from scratch, synthetic biology also involves the development of new technologies to control or modify existing biological metabolisms and processes [3], [4]. These engineered organisms or cells can be used for the production of pharmaceuticals, chemical compounds, biofuels, food, and so on.

In general, synthetic biology can be roughly divided into two subareas [5]: (1) *de novo* synthesis of an organism, such as designing and building a synthetic cell with an artificial genome from scratch; (2) re-engineering an existing organism, such as engineering metabolic pathways or constructing genetic circuits to achieve pre-designed functions in the target organism. Metabolic engineering can alter intrinsic metabolic pathways of an organism and/or add new metabolic pathways. Implementation of complex genetic circuits enables newly-designed functions within the target organism. In order to achieve these goals, genome writing technologies with different scales and complexities are needed. Genome writing can be achieved by *de novo* genome synthesis technology and genome editing technology [5]. *De novo* genome synthesis technologies are better suited for re-writing large-scale genomes with high complexity, whereas genome editing technologies are suitable for small-scale writing tasks on the limited genome loci. In this review, we mainly focus on genome writing by detailing relevant techniques and briefly summarize recent progresses and possible industrial applications. Finally, we elaborate on the current challenges and future directions.

## Genome writing is achieved through DNA synthesis and genome editing

### *De novo* synthesis and assembly technologies

*De novo* genome synthesis typically starts with assembly of synthetic single-stranded DNA oligos. These oligos can be annealed followed by PCR amplification to get the lowest dimension of double-stranded DNA fragments, which are usually less than 2 kilobases (Kb) in length. A complete artificial synthetic genome can then be obtained through stepwise DNA assembly. The assembly process is achieved at four scales: Kb-scale assembly, megabase (Mb)-scale assembly, chromosome-scale assembly, and genome-scale assembly.

Gibson assembly and Golden Gate assembly can be used for the Kb-scale assembly. The principle of Gibson assembly is similar to the process of homologous recombination, which is suitable for assembly of regular sequences but not highly-repetitive sequences. Gibson assembly is usually capable of piecing together up to five DNA fragments [6]. Golden Gate assembly uses a type IIS restriction enzyme based, restriction–ligation combined system for DNA assembly, which is suitable for all types of sequences including highly-repetitive ones, and usually up to nine DNA fragments can be assembled at one time [7]. One limitation of Golden Gate assembly is that it does not work when there are same type IIS restriction enzyme sites within the DNA fragments to be assembled.

Mb-scale assembly relies on the use of bacteria or yeast cells. Pre-assembled Kb-scale DNA fragments need to be transformed into these cells for a higher level of assembly. After several rounds of assembly, large DNA fragments in the range of a few hundred Kb to several Mb can be obtained. Several methods are available to assemble a Mb-scale DNA fragment, such as the switching auxotrophies progressively for integration (SwAP-In) [8].

Chromosome-scale assembly starts with pre-assembled large DNA fragments, *e.g.*, Mb-scale DNA donor. Usually, Mb-scale DNA fragments can be maintained as a yeast artificial chromosome within yeast cells. To complete the assembly, the donor yeast cell is typically fused with the host cell [9].

Genome-scale assembly can be accomplished by mating or microcell-mediated chromosome transfer (MMCT) [10]. Through chemically-induced micronucleation, artificial chromosomes are packaged with nuclear membrane to produce micronuclei. Cytoskeleton within the micronuclei is then disarranged by inhibitor to produce microcells containing separated chromosomes. After a microcell–host cell fusion, the artificial chromosome can be combined with the host genome [10].

### Genome editing technologies

In most cases, a *de novo* synthesized genome is not needed. Albeit on a much smaller scale, genetic manipulation of existing genomes via genome engineering is often sufficient. Targeted genome editing is typically achieved following two steps [11]. First, engineered endonucleases are used to make DNA double-stranded breaks (DSB) at the specific sites in the genome. As a consequence, intrinsic cellular DNA damage repair (DDR) pathways are activated to repair the DSBs and sometimes introduce insertions and/or deletions (InDels) at the target sites. To date, several types of endonucleases have been exploited, including zinc finger nucleases (ZFNs), transcription activator-like effector nucleases (TALENs), and clustered regularly-interspaced short palindromic repeats (CRISPR)/CRISPR-associated protein (Cas) systems, among others [12]. They all have a high degree of targeting flexibility and can efficiently make DSBs in targeted genome loci. Regardless, there are significant differences among these three systems. ZFNs and TALENs are engineered proteins that target specific DNA sequences through protein-DNA interaction. As a result, ZFNs and TALENs are usually difficult to design and construct. In contrast, the CRISPR/Cas system relies on the simple design and synthesis of an RNA molecule complementary to the DNA target sites.

There are two main mechanisms for DSB repair, *i.e.*, non-homologous end joining (NHEJ) and homology-directed repair (HDR) [13]. NHEJ repair does not require a donor DNA, and often InDels are introduced at the target sites. HDR requires a donor DNA to facilitate precise genome modification at the cleavage sites. In addition, repurposing of engineered nucleases can also enable inactivation of their endonuclease activity while retaining the DNA binding capabilities. When fused with functional moieties, *e.g.*, epigenetic modification enzymes, these tools can be harnessed for epigenetic modification at target genome sites [14], [15]. As another example, when fused with a base editor, these tools can covert a specific nucleotide to another without introducing a DSB [16].

Combining *de novo* genome synthesis and genome engineering may constitute a superior approach for assembling a large artificial genome. For example, existing genome can be edited first and then used as the starting material for *de novo* genome assembly. Furthermore, available genome editing tools may facilitate the integration of large DNA fragments.

## Genome writing project further human understanding of biological processes

### Understand gene essentiality

Undoubtedly, the most ambitious genome-writing project is the generation of a synthetic cell with a functional synthetic genome. The need to design an artificial genome leads to a fundamental question: what is gene essentiality? Cellular pathways are either essential or non-essential [17]. Essential pathways are mostly tasked with basic and fundamental cellular functions that support cell/organism viability, whereas non-essential pathways are often cell or species-specific. In order to adapt to the external environment, cellular metabolic pathways undergo extensive evolutions, resulting in the development of a series of complex secondary metabolic mechanisms. It has been a long-standing interest and effort to study the biological processes and metabolic pathways of natural cells and organisms. Grateful to the accumulating knowledge, nowadays we are able to create a cell with a minimal genome by removing non-essential biological processes and metabolic pathways [18]. These cells constitute an engineering platform where designer cells with desired functions can be engineered by adding specific metabolic pathways and genetic circuits to the minimal genome. For the first proof-of-concept, in 2016, Hutchison et al. reported a synthetic *Mycoplasma mycoides* genome from the JCVI-syn3.0 project, which represents an important milestone in the synthetic biology field. Aimed to build a synthetic minimal genome, they maximally deleted the non-essential genomic DNA from the original genome, and the synthesized genome only has a size of 531 Kb and contains 473 genes [18]. To our knowledge, this is the first and only completed synthetic chassis cell with a quasi-essential genome.

### Alter genetic encoding/decoding rules

Genome writing does not have to follow the existing rules of genetic encoding and decoding, thereby holding the potential to create new life forms that do not exist in nature [19]. Although different organisms differ vastly in many aspects, their genomes are encoded by the same four nucleotides, and their proteins are assembled with the same 20 types of amino acids. Synthetic organisms, on the other hand, may be designed to incorporate additional types of nucleotides into their genomes and non-canonical amino acids into their proteins. By altering the codon rules and using modified tRNAs, new proteins containing non-canonical amino acids can be obtained. In 2013, Lajoie et al. replaced the TAG stop codons with the TAA stop codons and reassigned TAG codon for non-canonical amino acids like *p*-acetylphenylalanine in *Escherichia coli* MG1655 [20]. In 2017, Zhang et al. incorporated unnatural base pairs into DNA and non-canonical amino acids into green fluorescence protein using specific tRNAs [19]. These studies have greatly expanded the repertoire for central dogma including DNA replication, transcription, and translation, thereby paving the way for creating a variety of artificial organisms with special features.

### Build artificial life with synthetic/chimeric genome

*De novo* design of an artificial genome holds promise for gaining novel insights into cellular processes and metabolic pathways. In order to make an artificial cell with a functional synthetic genome, several rounds of the design-build-test (DBT) processes are needed. The DBT processes may help to discover and understand the mechanisms and processes that are otherwise not accessible with traditional means. Different organisms vary greatly in their composition of genomes and proteomes, as well as their metabolic processes and cellular functions. However, they do share many common features in basic cellular processes [17]. For example, all cells, albeit from different organisms, have common processes for energy metabolism, DNA replication, DDR, RNA transcription, protein translation, *etc*. However, in different cells or organisms, these processes are likely regulated by different genes and proteins, which often share sequence homologies. *De novo* synthesis and assembly of different genomes will help gain insights into the conservation and divergence, as well as the crosstalk between genetic elements and proteins from different organisms.

In recent years, several genomic DNA synthetic projects have been completed or underway, including viral, prokaryotic, eukaryotic, and mammalian genome (Table 1) [8], [18], [21], [22], [24], [25], [26], [27], [28], [29]. Among them, the humanized mouse project represents an important advance. Researchers used human immunoglobulin loci to replace the corresponding immunoglobulin loci in the mouse genome. The obtained humanized mice have a functional immune system and are able to produce humanized antibodies [23], [24].

**Table 1:** **Summary of synthetic genomic DNA projects**

**No.**	**Year**	**Species**	**Classification**	**Project name**	**Scale**	**Ref.**
1	2002	*Poliovirus*	Virus	N/A	7.5 Kb	[21]

2	2010	*Mycoplasma mycoides*	Bacteria	JCVI-syn1.0	1.08 Mb	[22]

3	2011	*Saccharomyces cerevisiae*	Fungi	Sc2.0_synIX-R	91 Kb	[8]
				Sc2.0_semi-synVI-L	30 Kb	[8]

4	2014	*Homo sapiens*	Mammal	N/A	1.42 Mb	[23]

5	2014	*Homo sapiens*	Mammal	N/A	2.7 Mb	[24]

6	2016	*Mycoplasma mycoides*	Bacteria	JCVI-syn2.0	576 Kb	[18]
				JCVI-syn3.0	531 Kb	[18]

7	2017	*Saccharomyces cerevisiae*	Fungi	Sc2.0_synXII	976 Kb	[25]

8	2017	*Saccharomyces cerevisiae*	Fungi	Sc2.0_synVI	242 Kb	[26]

9	2017	*Saccharomyces cerevisiae*	Fungi	Sc2.0_synII	770 Kb	[27]

10	2017	*Saccharomyces cerevisiae*	Fungi	Sc2.0_synX	707 Kb	[28]

11	2017	*Saccharomyces cerevisiae*	Fungi	Sc2.0_synV	536 Kb	[29]

## Advances in genome writing drive the birth of commercial applications

Cellular processes such as metabolism are largely controlled by genome. Genome writing technologies can change genome information at a large or small scale, which enables the design and engineer of organism catering to specific applications.

**Figure 2:**
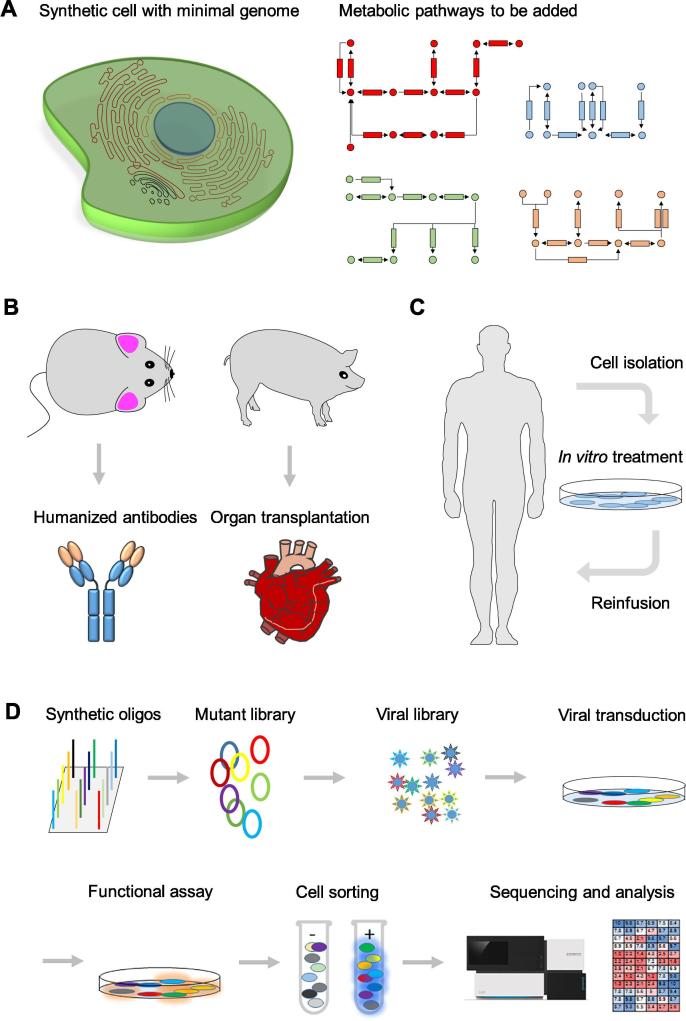
**Examples of genome writing based applications** **A.** Designer cells with customized metabolism. A pre-built cell with the minimal genome can be used to add relevant metabolic pathway(s) and genetic circuits for different applications. **B.** Making humanized animal models. Humanized mice can be used for therapeutic antibody production and humanized pigs can be used for organ transplantation. **C.** Using genetically-engineered cells for human disease treatment. First, stem cells or precursors are isolated from the patient, followed by genetic manipulation *in vitro*. Subsequently, those engineered cells are reinfused into the patient to cure disease. **D.** Schematic diagram of deep mutational scanning process. A synthetic mutant library is constructed and packaged as viruses. After transduction, positively-transduced cells are treated by a gene-specific functional assay. This assay should be able to separate the cells infected with the pathogenic variants from the cells infected with the neutral variants. Through fluorescence-activated cell sorting, those cells can be separated into two groups. One group of cells is primarily infected with pathogenic variants, while the other group of cells is predominantly infected with neutral variants. Later, variants are amplified from both groups of cells and then subjected to next-generation sequencing. Mutational effects are evaluated based on complicated mathematical models. All variants will have evaluation scores which can be used to make a visual diagram, *e.g.*, heatmap.

Here, we list some examples on how to use genome writing technologies to develop useful products or therapeutics (Figure 2). (1) The synthetic cell with a minimal genome and customized metabolism pathway(s) [18]. The synthetic cell is designed to only possess necessary fundamental metabolism in order to reduce nutrient loss and promote the synthetic efficiency of target products. For different applications, relevant metabolic pathway(s) can be incorporated into the genome of these synthetic cells. For example, to produce biofuels, ethanol biosynthesis pathway can be added [30], [31]; to produce antimalarial pharmaceutical compounds, artemisinin biosynthesis pathway may be added [32]. (2) Gene therapy and cell therapy [33], [34], [35], [36]. Stem cells are isolated from the patient with genetic defects. Gene editing is then employed to correct genetic mutations *in vitro*. Finally, the genetically-corrected cells are transplanted back to the patient to cure the disease. In addition to repair defective genes, more sophisticated genome manipulations, *e.g.*, incorporating genetic circuits, has been used for cancer immunotherapy. In 2017, Nissim et al. reported using a genetic AND logic circuit to direct immune cells to specifically target and kill cancer cells [36]. (3) Generation of humanized animal models [23], [24], [37]. Using genome editing, we can humanize animal genomes for different applications. For example, humanized mice are used to develop humanized antibodies. Humanized pigs may offer a solution to solve worldwide shortage of organ donors by providing immune-compatible pig organs for human transplantations in the future. (4) High-throughput functional study of human genetic variants [38], [39]. The costs of genomic sequencing are dropping rapidly, and vast amounts of genomic data are produced. However, due to the lack of annotations of the vast majority of human variants, it is difficult to extract useful information from personal genome sequencing data. The rapid development of genome writing technologies has led to a significant reduction in the cost of large-scale DNA synthesis. Using techniques such as deep mutational scanning combined with high-throughput sequencing, we are able to perform high-throughput functional studies of human variants [38]. This will greatly advance the development of personal genome sequencing projects and facilitate precision medicine applications.

## Challenges and prospects

Since the beginning of the 21st century, rapid progresses have been made in the synthetic biology field. However, there still remain many challenges, which require continued investments and efforts.

(1) Further understanding of the cellular/biological processes and metabolism pathways is needed. Understanding of the natural biological processes and metabolism pathways is a prerequisite to the development of any new synthetic biology designs and applications. There are more than eight million types of different species living on Earth. Among them, many are complex multicellular organisms that have a multitude of cell types with different compositions and functions. These cells share common fundamental metabolisms, but also differ in the specific metabolic pathway(s). What are the factors involved? What are the relations between these factors? Lots of work needs to be done to answer these questions.

(2) Technically, the cost of genome synthesis needs to be reduced and the efficiency increased. At present, implementing a large-scale synthetic genomic project is still costly and time-consuming. For small-scale genome editing, the current techniques are still inefficient in HDR-based precision editing. Genome editing based on the customizable nucleases relies on the intrinsic cellular DNA DSB repair mechanisms. Due to the difficulties of precisely controlling the DNA repair processes, the efficiency for error-free repair is inherently low. In addition, the off-target effects are also an important concern that needs to be further addressed.

(3) There are bioethics and biosafety concerns [40], [41]. Synthetic biology has made it easy to synthesize human-animal chimeric genomes. A human-animal chimera can be made by incorporating large amounts of human genomic fragments into an animal genome, or *vice versa*. As time goes by, it will become increasingly difficult to define whether such kinds of chimeras are human or animal. In addition, it has been reported that synthetic viruses have the same infectivity compared to their natural counterparts [21]. Criminals or terrorists may use synthetic pathogens to engage in criminal activities that endanger public safety. Therefore, we must take measures to ensure that synthetic biology technologies are not indiscriminately used to prepare toxic, infectious, and other harmful organisms or biological materials.

In general, synthetic biology marks a new era in the biological field. Different from physics and chemistry, there do not exist simple and clear disciplines to follow in biology area when developing applications. Basically, all species are black boxes with extremely complex structures and functions. Fully understanding the laws is the premise of all applications for every discipline. In the past century, the biological studies have undergone a rapid development, which tremendously promotes our understanding of organismal heredity and development. All these have laid an important foundation for the birth of synthetic biology. In the future, with a combination of genome writing technologies, genetic circuit technologies, new genetic encoding and decoding technologies, *etc.*, we might be able to create new species or resuscitate extinct species according to human needs.

## Competing interests

The authors declare no competing interests.
